# A Reagent and Virus Benchmarking Panel for a Uniform Analytical Performance Assessment of N Antigen-Based Diagnostic Tests for COVID-19

**DOI:** 10.1128/spectrum.03731-22

**Published:** 2023-05-11

**Authors:** Allison Golden, Jason L. Cantera, Lorraine Lillis, Thanh T. Phan, Hannah Slater, Edwin J. Webb, Roger B. Peck, David S. Boyle, Gonzalo J. Domingo

**Affiliations:** a PATH, Seattle, Washington, USA; University of Arizona/Banner Health

**Keywords:** SARS-CoV-2, rapid antigen diagnostic test, rapid diagnostic test, COVID-19, screening, nucleocapsid antigen, spike antigen, genome equivalent, PCR, rapid antigen test

## Abstract

Rapid diagnostic tests (RDTs) that detect antigen indicative of severe acute respiratory syndrome coronavirus-2 (SARS-CoV-2) infection can help in making quick health care decisions and regularly monitoring groups at risk of infection. With many RDT products entering the market, it is important to rapidly evaluate their relative performance. Comparison of clinical evaluation study results is challenged by protocol design variations and study populations. Laboratory assays were developed to quantify nucleocapsid (N) and spike (S) SARS-CoV-2 antigens. Quantification of the two antigens in nasal eluates confirmed higher abundance of N than S antigen. The median concentration of N antigen was 10 times greater than S per genome equivalent. The N antigen assay was used in combination with quantitative reverse transcription (RT)-PCR to qualify a panel composed of recombinant antigens, inactivated virus, and clinical specimen pools. This benchmarking panel was applied to evaluate the analytical performance of the SD Biosensor Standard Q COVID-19 antigen (Ag) test, Abbott Panbio COVID-19 Ag rapid test, Abbott BinaxNOW COVID-19 Ag test, and the LumiraDx SARS-CoV-2 Ag test. The four tests displayed different sensitivities toward the different panel members, but all performed best with the clinical specimen pool. The concentration for a 90% probability of detection across the four tests ranged from 21 to 102 pg/mL of N antigen in the extracted sample. Benchmarking panels provide a quick way to verify the baseline performance of a diagnostic and enable direct comparisons between diagnostic tests.

**IMPORTANCE** This study reports the results for severe acute respiratory syndrome coronavirus-2 (SARS-COV-2) nucleocapsid (N) and spike (S) antigen quantification assays and their performance against clinical reverse transcription (RT)-PCR results, thus describing an open-access quantification method for two important SARS-CoV-2 protein analytes. Characterized N antigen panels were used to evaluate the limits of detection of four different rapid tests for SARS-CoV-2 against multiple sources of nucleocapsid antigen, demonstrating proof-of-concept materials and methodology to evaluate SARS-CoV-2 rapid antigen detection tests. Quantification of N antigen was used to characterize the relationship between viral count and antigen concentration among clinical samples and panel members of both clinical sample and viral culture origin. This contributes to a deeper understanding of protein antigen and molecular analytes and presents analytical methods complementary to clinical evaluation for characterizing the performance of both laboratory-based and point-of-care rapid diagnostics for SARS-CoV-2.

## INTRODUCTION

Diagnostic tools are essential for surveillance and control of the COVID-19 pandemic (1). While reverse transcription (RT)-PCR from a nasopharyngeal or nasal swab is the gold standard for confirmation of infection with severe acute respiratory syndrome coronavirus-2 (SARS-CoV-2), the complexity of such tests requires sophisticated laboratory systems and imposes logistical challenges for its effective use in scenarios requiring either a fast time to result or where laboratory systems are not robust. Rapid diagnostic tests (RDTs) designed to detect viral antigens, primarily the nucleocapsid protein (N) antigen, hold promise for testing in settings in which RT-PCR cannot be implemented and as both time- and cost-saving measures for frequent testing and entry points (1–3).

The earliest-to-market RDTs were tested in clinical studies to assess their performance and utility. The results of these clinical studies are informative in terms of clinical performance within the context of the studies conducted, but they also highlight the variability in clinical performance as driven by the study design, target population, and other study-specific factors (4–6).

There is need for a performance assessment of these tests using more controllable sample characteristics than prospective clinical studies and that can be performed in multiple laboratories, the results of which could enable more direct comparison of performance across different RDTs (7). If the results of this assessment can be linked to clinical data, they may also be indicative of clinical performance. Currently, assessments of analytical performance have been expressed primarily through comparison to SARS-CoV-2 viral RNA in terms of cycle thresholds (Ct) or to cultured virus infective units, with an increasing correlation to viral copy number quantification to aid in standardization of results (7–13). Complementary to these efforts, this article presents a quantitative open-platform assay for the N and spike (S) antigens, a comparison of genome equivalents (GEs) to N and S antigen concentration from clinical samples, a panel of reagents with which to assess the performance of the RDTs representing multiple sources of target analyte protein, and the results from assessment of four different emergency use-authorized (EUA)/emergency use-licensed (EUL) COVID-19 rapid antigen diagnostic tests.

## RESULTS

### Analytical performance for the SARS-COV-2 N and S proteins.

The SARS-CoV-2 N antigen immunoassay on the Meso Scale Discovery (MSD) platform had an average limit of detection (LOD) of 0.45 pg/mL (0.25 to 0.93 pg/mL range), a lower limit of quantification (LLOQ) of 3.2 pg/mL, and an upper limit of quantification (ULOQ) of 50 ng/mL. The SARS-CoV-2 S antigen immunoassay had an average LOD of 6.2 pg/mL (2.1 to 9.0 pg/mL range), an LLOQ of 80 pg/mL, and a ULOQ of 250 ng/mL. Both assays were nonreactive or below detection limits for panel diluents, transport media, and common human coronavirus lysates OC43 and 229E.

### Distribution of N and S antigens in clinical samples.

**FIG 1:**
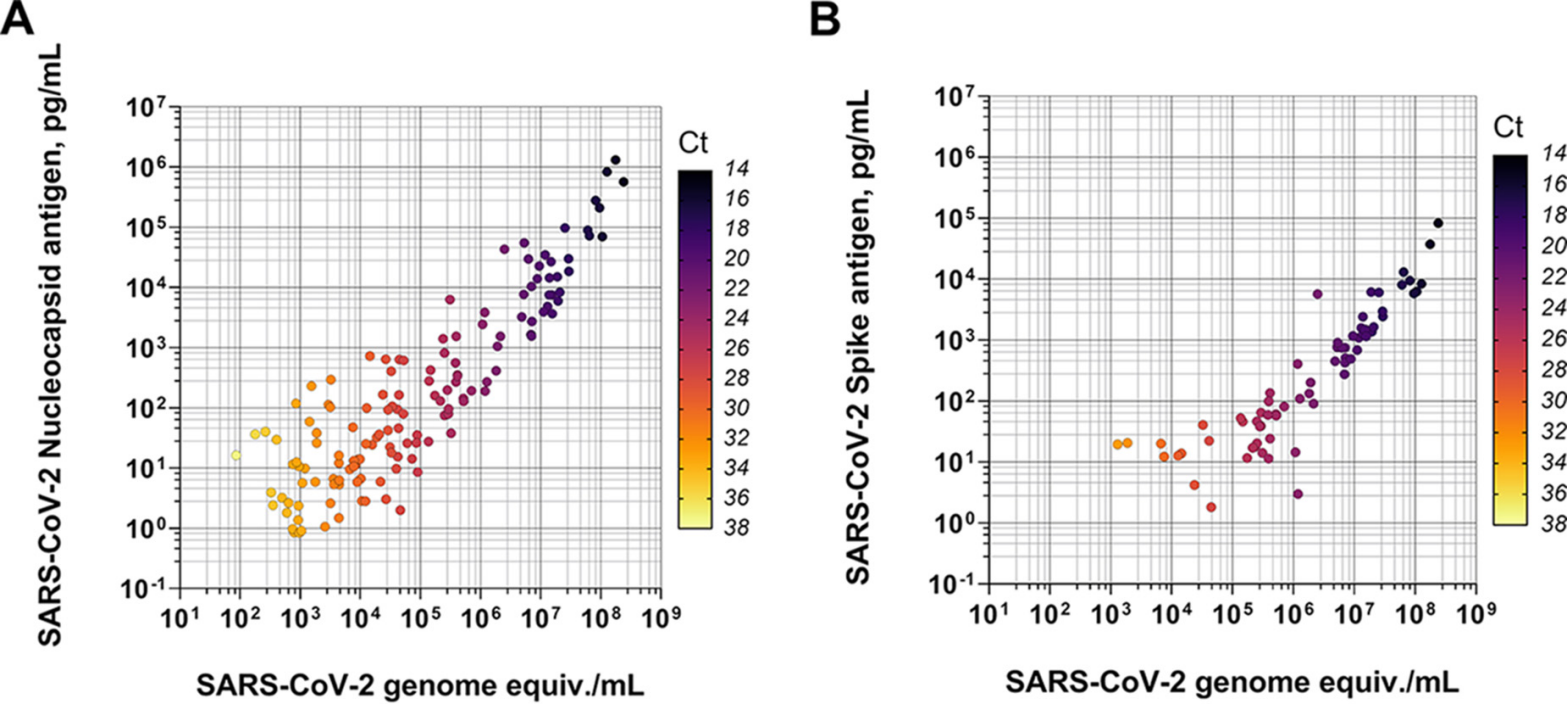
(A) Correlation between nucleocapsid (N) antigen concentration (pg/mL) and genome equivalents (copies N gene/mL). Mean cycle threshold (Ct) values are color coded to provide an indication of the corresponding Ct values. (B) Correlation between spike (S) antigen concentration (pg/mL) and genome equivalents copies N gene/mL. Mean Ct values are color coded to provide an indication of the corresponding Ct values. SARS-CoV-2, severe acute respiratory syndrome coronavirus-2.

**FIG 2:**
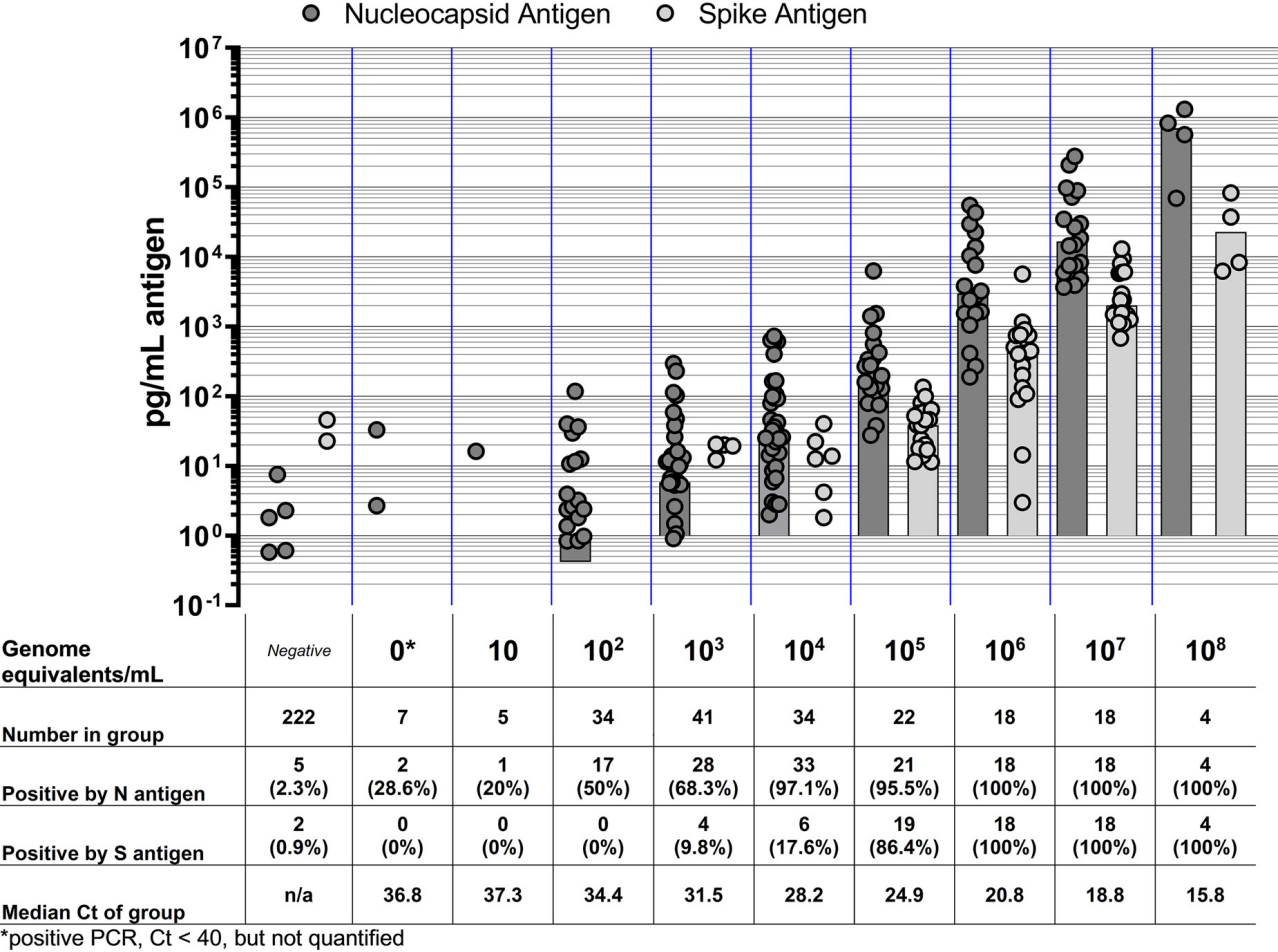
Antigen concentration over a range of SARS-CoV-2 genome equivalents detected by Meso Scale Discovery antigen quantification. Characteristics of groups of viral genome equivalent concentrations are shown in the table below the graph. Bars indicate the median of the group of antigen-positive points shown. Ct, cycle threshold; N, nucleocapsid; S, spike.

All 200 presumed positive samples and 100 of the 205 negative samples were analyzed by quantitative reverse transcription (qRT)-PCR for SARS-COV-2. Here, 182 samples were confirmed positive by qRT-PCR, and 99 of the negatives were confirmed negative, with one repeatedly testing positive by qRT-PCR. The remaining negatives were assigned as qRT-PCR-negative based on previously assigned qRT-PCR results, resulting in 183 PCR-positive samples and 222 PCR-negative samples. N and S antigen quantification was conducted on all 405 specimens. A positive correlation was found between the N antigen concentration and GEs (Fig. 1A and B). The N antigen assays showed a high percentage of positive agreement (>95%) for specimens containing 10^4^ or more GEs/mL SARS-CoV-2 (Fig. 2). The percent positive agreement progressively dropped with decreasing concentrations of GEs. For the S antigen, the percent positive agreement was 86.4% at 10^5^ GEs/mL, with a sharp drop in agreement for lower concentrations. For all samples for which N antigen was within the LOQ of the assay (*n* = 120), the mean and median N antigen per GE observed were 12.7 fg/GE and 1.5 fg/GE N antigen (range, 0.1 to 204.2 fg/GE). For concentrations of SARS-CoV-2 GEs less than 10^4^ GEs/mL, the quantity of N antigen varied more widely for positive samples within the LOQ, with a trend toward higher N antigen per GE. For all samples for which S antigen was within the LOQ of the assay (*n* = 41), the mean and median S antigen per GE observed were 0.2 fg/GE and 0.1 fg/GE S antigen (range, 0.04 to 2.3 fg/GE).

### Benchmarking panels.

Benchmarking panels were prepared to span concentrations of N antigen corresponding to LODs expected in rapid tests following dilution into the extraction buffer. As most RDTs detect only the N antigen, S antigen analysis was not included in the panel. HEK293-expressed and Escherichia coli-expressed recombinant proteins were prepared in both buffer and negative swab pool dilution matrices, and the linear fit of measured concentration compared to target concentration was greater than 0.98 over the range of the panels of 0.2 to 50 ng/mL.

### Quantification of inactivated virus and clinical dilutions, compared to genome equivalents.

**FIG 3:**
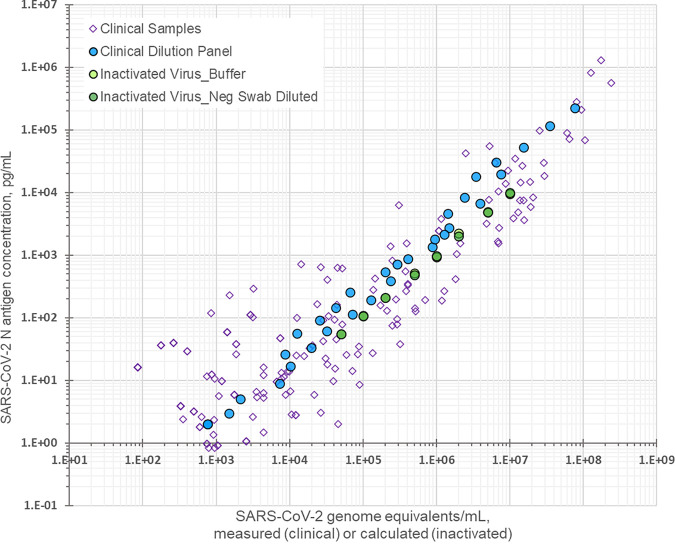
The relationship between nucleocapsid (N) antigen concentration and genome equivalents for panels of diluted clinical specimens and inactivated virus. Blue circles indicate the clinical pool dilution series. Light green and dark green circles indicate BEI Resources irradiated virus lot 7003588 in buffer and negative swab pool, respectively.

Although all panels were prepared from a single lot of inactivated SARS-CoV-2 (BEI Resources), comparisons between two lots showed that for each lot, the per-calculated GE concentration of N antigen had a median of 1.0 fg/GE (range, 0.51 to 1.1 fg/GE) over the dilutions. In contrast, compared to 50% tissue culture infective dose (TCID_50_), the median amount of N antigen per TCID_50_ in the dilutions was 5,820 fg/TCID_50_ for BEI Resources lot 70033322 and 668 fg/TCID_50_ for lot 70035888. Both irradiated virus and clinical pool samples behaved similarly, even in terms of the per GE concentration of N antigen (Fig. 3). For the clinical specimen pool dilution series, the median per GE quantity of N antigen was 2.4 fg/GE (range, 1.2 to 5.3 fg/GE).

### Benchmarking of rapid antigen detection tests for SARS-CoV-2.

Four RDTs were evaluated with the benchmarking panels: Abbott BinaxNOW COVID-19 antigen (Ag) card test, Abbott Panbio COVID-19 Ag rapid test device, LumiraDx SARS-CoV-2 Ag test, and SD Biosensor Standard Q COVID-19 Ag home test. The tests were anonymized using identification numbers RDT 1 through RDT 4, in no particular order, for comparative presentations of the results.

### Reactivity of panel types.

**FIG 4:**
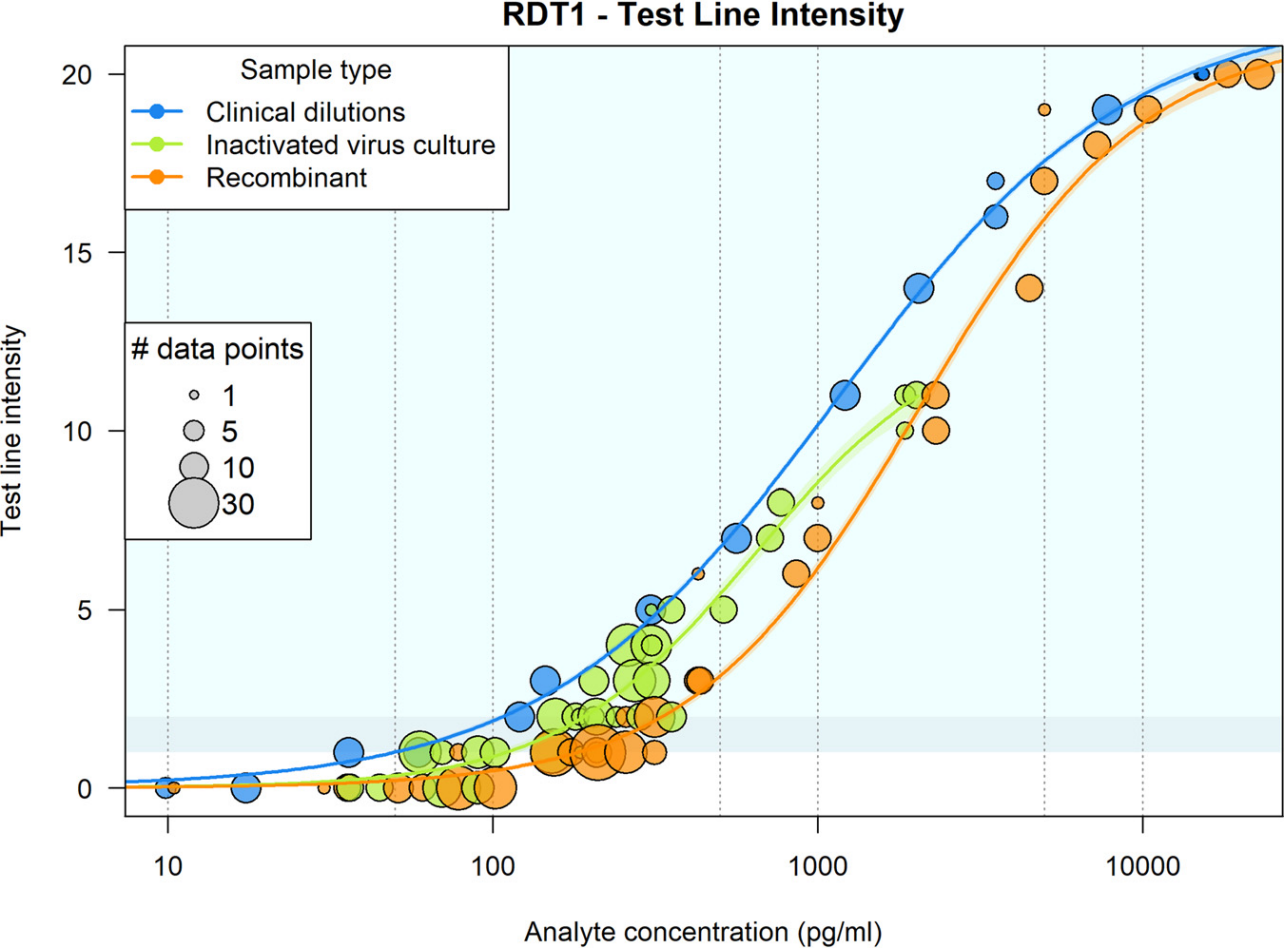
Illustrative panel subset analysis for SARS-CoV-2 rapid diagnostic test 1. Circled positions indicate the replicates with a given test line intensity result for the concentration of antigen (analyte) panel added. Each panel subset has been given a different color code: blue for clinical specimen pool, green for inactivated virus, and orange for recombinant antigen. The sizes of the circles indicate the number of replicates supporting each data point. Test line intensity is shown on a scale of 0 (negative) and 1 through 20, representing from least to most intense visible test line of positive results. RDT, rapid diagnostic test.

**FIG 5:**
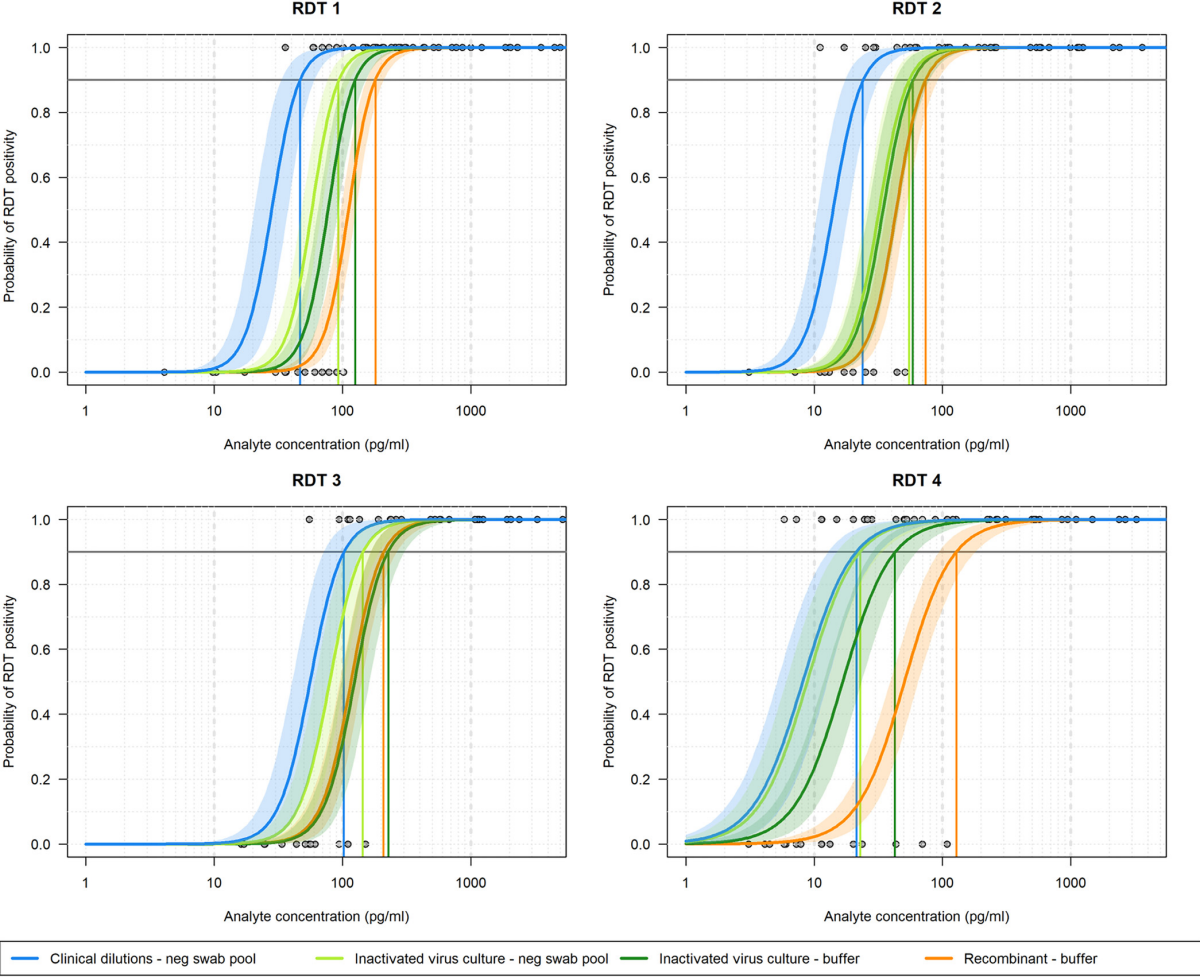
Probability of detection against antigen concentrations for the four antigen detection rapid diagnostic tests per benchmarking panel type and diluent matrix and probability of detection of positive for the different benchmarking panel member types for each test in each panel. Blue lines indicate clinical specimen dilution; green lines represent inactivated virus; and orange lines represent recombinant protein. Credible intervals (95%) are indicated in matched-color shading and vertical lines mark positions of 90% probability. RDT, rapid diagnostic test.

For all RDTs, a dose-responsive visual test line intensity or output signal was observed, relative to N antigen concentration. An illustrative example is given in Fig. 4 for RDT 1. Recombinant proteins from mammalian and E. coli-based expression systems produced a strong dose-response signal on the RDTs and were similarly reactive. Greater reactivity was observed with both inactivated viral culture and diluted clinical positives, based on the final concentration of N antigen. Binary positive/negative results were used to model the probability of detection at different antigen concentrations and compare the detection limit across RDTs. This was performed for all four tests (Fig. 5). All tests performed best against the clinical specimen pool dilutions in terms of 90% probability of detection and then differentially against the different panel members. RDTs had lower reactivity to the inactivated virus when it was diluted into the buffer diluent versus negative swab pool diluent (Table 1).

**TABLE 1:** Ninety percent probability of detection (95% credible interval) of N antigen final concentration for benchmarking panel categories^*a*^

Test	Concn of N antigen (final concn added to test) with 90% probability of detection, pg/mL				
	Diluted in negative swab pool		Diluted in buffer		Specificity and diluent control panel
	Clinical positive panel	Inactivated virus panel	Inactivated virus panel	Recombinant protein panel (E. coli and mammalian expressed)	
RDT 1	46.8 (33.1 to 60.3)	93.3 (75.9 to 114.8)	125.9 (102.3 to 151.4)	182.0 (154.9 to 208.9)	Not detected
RDT 2	24.0 (17.4 to 31.6)	55.0 (40.7 to 72.4)	58.9 (42.6 to 79.4)	74.1 (58.9 to 91.2)	Not detected
RDT 3	102.3 (70.8 to 141.3)	144.5 (104.7 to 195.0)	229.1 (166.0 to 302.0)	208.9 (166.0 to 263.0)	Not detected
RDT 4	21.4 (12.9 to 32.4)	22.9 (15.1 to 33.9)	42.7 (28.8 to 63.1)	128.8 (93.3 to 182.0)	Not detected

a RDT, rapid diagnostic test.

### Comparative benchmarking results.

**FIG 6:**
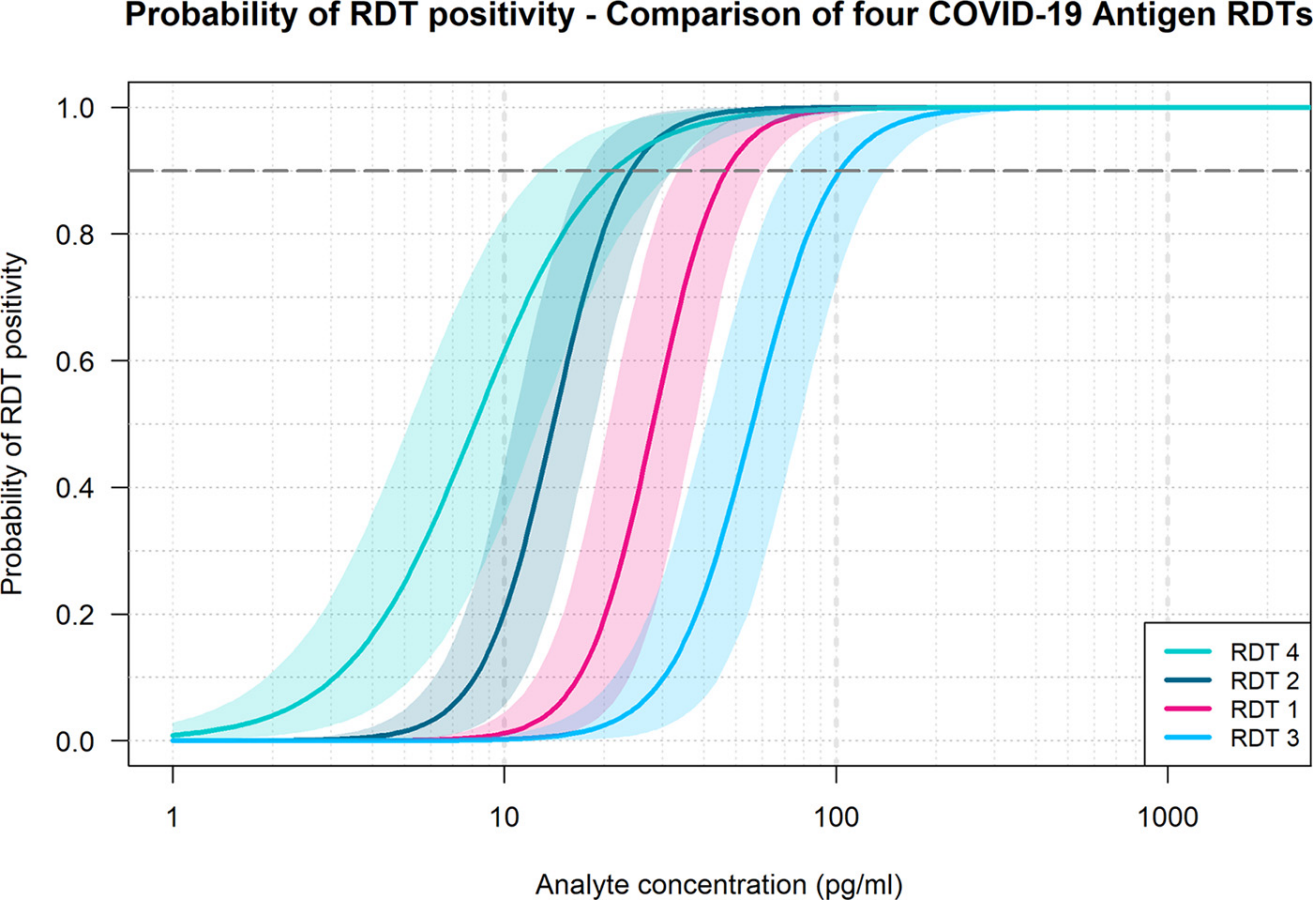
Probability of test positivity versus final nucleocapsid (N) antigen (analyte) concentration added to test for clinical positive dilutions. The four curves indicate the probability of test positivity for each rapid antigen test product. The shaded lines indicate 95% credible intervals. RDT, rapid diagnostic test.

The clinical specimen pool dilution panels were plotted for all four tests for comparison of analytical performance of the RDTs (Fig. 6). The modeled 90% probabilities of detection were found to be 47, 24, 102, and 21 pg/mL of final concentration of N antigen added to test, for RDTs 1 through 4, respectively (Table 1).

### Simulating clinical performance.

One limitation of benchmarking is that the results are interpretable as a concentration inherently dependent upon assay configuration and the input volumes of the analyte. Interpretation of the final concentration of incoming analyte, as diluted into the assay extraction buffer, allowed normalization across RDTs but created the challenge of direct comparison to qRT-PCR values. If an identical swab were diluted into transport media (typically around 3 mL) and into RDT extraction buffer (typically around 300 μL), the volume difference could create a 10-fold disparity in analyte concentration from the swab. Using this dilutional difference and the RDT limits of detection, detectability of clinical samples by RDTs was modeled. Limits of detection determined by the 90% cutoff were compared against the clinical sample set antigen concentrations, with the assumption that the extracted material present in the PCR would instead be fully present in the extraction buffer for the rapid test, thus simulating a paired swab experiment. The detection limits based on the concentration of N antigen added to the test were found to affect the number of samples predicted to be detectable.

**FIG 7:**
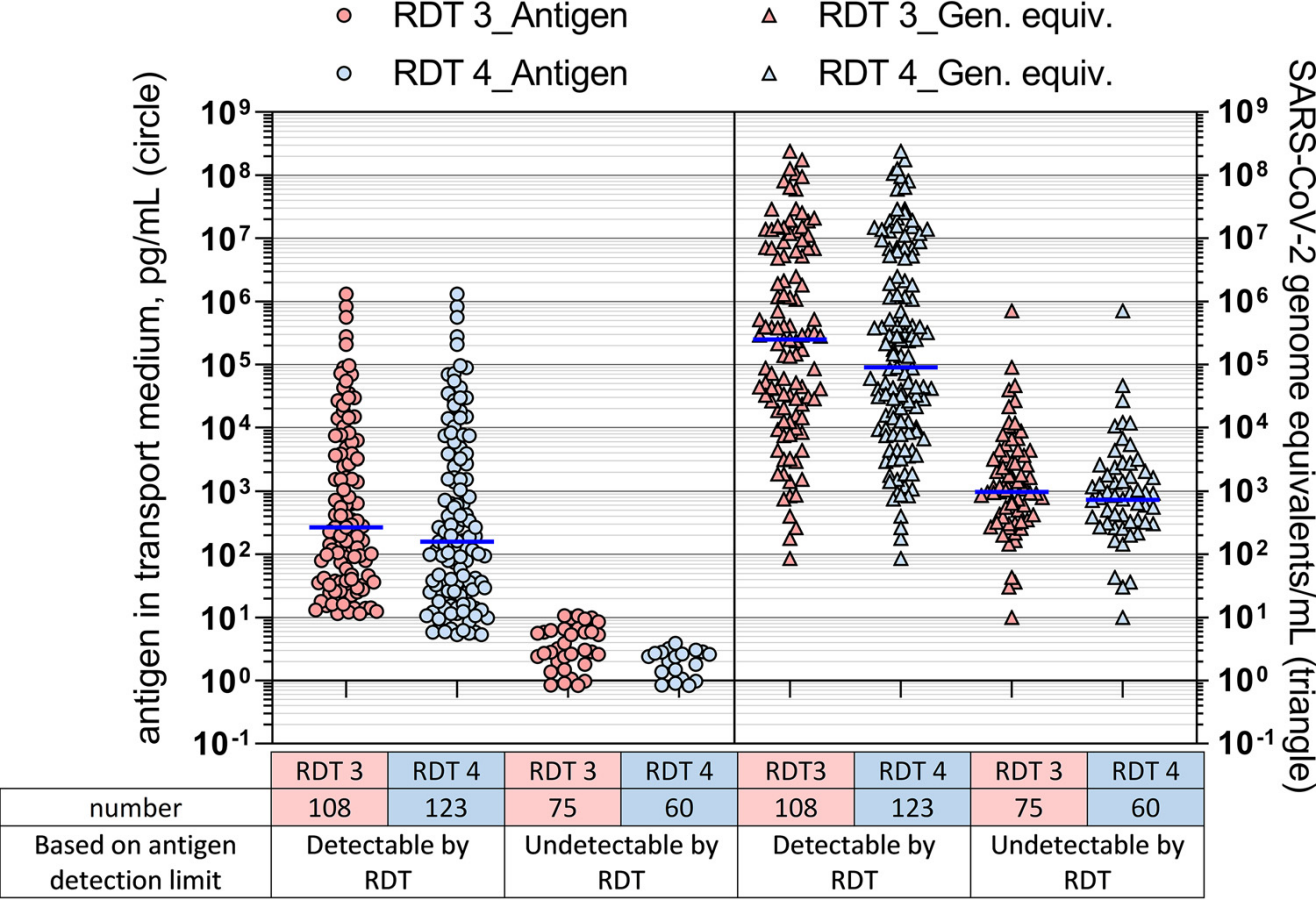
The predicted rapid diagnostic test (RDT) positivity from clinical samples based on their nucleocapsid antigen detection limit. The results are for RDT 3 (pink shading) and RDT 4 (blue shading), shown as antigen concentration versus positivity (circle) or viral genome equivalent/mL versus positivity (triangle).

RDTs 3 and 4, which had the highest and lowest analytical detection limits, respectively, based on final concentration, were compared in this simulation. Overlap in N antigen-detectable and -undetectable concentrations of GEs (Fig. 7) was observed due to the spread of antigen concentration per GE relationship, but the median GEs/mL between the detectable and undetectable differed by about 2 orders of magnitude for all RDTs.

## DISCUSSION

An open-platform assay was developed and described to quantify the N and S antigens in SARS-CoV-2-infected clinical specimens on the MSD platform. The assays showed good performance against RT-PCR-confirmed cases and a panel of negative specimens. Quantification of both the N and S antigens in specimens with associated viral load values showed a positive but not perfect correlation. As anticipated, a higher N antigen concentration was observed per GE in comparison to S antigen concentration, which has also been shown in plasma (14, 15). These results observed correlations of antigen concentration with genome copy number (9, 16, 17). It also highlights the rationale to utilize the N antigen as target analyte for most rapid tests on the market, as N is in greater abundance than S per viral particle.

The antigen assay combined with the qRT-PCR was used to pedigree a panel of reagents designed to benchmark N antigen RDTs. The benchmarking panel consisted of a dilution series of recombinant N antigens, expressed in both prokaryotic and eukaryotic expression systems, two sources of inactivated virus, and a clinical specimen pool. Four widely used tests, either cleared by EUL or EUA, were evaluated against the panel: SD Biosensor Standard Q COVID-19 Ag home test, Abbott COVID-19 Ag rapid test device, Abbott BinaxNOW COVID-19 Ag card test, and LumiraDx SARS-CoV-2 Ag test. E. coli-expressed recombinant N antigen behaved similarly to N antigen expressed in mammalian cells on the N antigen assay. Proteins from both expression systems were included in order to compare reactivities since bacterially expressed proteins may be utilized in test development or for rapid expression of sequence variants. Based on the data from this study, possible differences in post-translational modification between the two expression systems did not seem to greatly alter the reactivity of the two types of recombinants for any of the tests evaluated. For future use of recombinant protein in a SARS-CoV-2 benchmarking panel, the ideal expression system would be mammalian to reflect the human host in clinical diagnosis. Inactivated virus contained N antigen concentration per viral GEs within the same range as observed in clinical samples. Comparison of the relationship between N antigen concentration and GEs versus TCID_50_ across two lots from BEI Resources suggests that GEs are more reflective of the anticipated N antigen concentration.

While all tests evaluated are qualitative tests, clear correlations were seen between signal out/test line intensity versus analyte concentration. While only binary data were considered for limit of detection analysis in this study, analysis of intensity can provide more granular data for quality evaluation of test-test variability and for monitoring of capture/detector reagent balances in a test. Curve fits of test positivity versus antigen concentration for the four tests reveal the range of reactivities against the different panel components and are agnostic to the intensity scale of the test. Consistently, all tests showed improved LOD to the clinical specimens over the other N antigen sources (inactivated virus and recombinant antigen). RDT 4 showed an improved response to negative swab diluent over buffer and highlights the need for comprehensive and test-specific data with a variety of materials before conclusions can be made. Understanding the reactivity against inactivated virus and recombinant protein is valuable, as these can be readily expressed, noninfectious sources of N antigen for emerging virus strains, for which there may be a concern for sequence-dependent false negativity (18–21).

The 90% probability of detection for the clinical specimen pool ranged from 20 to 100 pg/mL across the four tests. The 90% probability of detection model has been used to describe rapid test data detection limits in other studies (22, 23) and uses a logistic regression fit to dose-response binary data generated using standardized panels designed to span detection limits rather than requiring custom dilutions for each test (24). This model type can yield similar predictions to probit analysis with an intuitive output and is generally more robust to outlier values. The simulated paired swab results generated by applying analytical detection limits to clinical samples was in alignment with observed clinical performance evaluations, indicating that RDTs detect a high percentage of infections with viral loads associated with Ct values less than 30 (25, 26).

While the analytical performance measured through benchmarking may be strongly indicative of the clinical performance of the tests, it cannot be correlated directly to final performance. Some of the factors that will influence the final performance are (i) relative efficiencies for absorption and release of N antigen by the manufacturer’s specific nasal swab and elution buffer, (ii) the dilution factor and original specimen equivalents loaded on to the test after all processing is conducted, and (iii) differing sensitivities of the test to the circulating SARS-CoV-2 strains in the population being sampled. Although RDTs showed the best analytical performance against the diluted clinical specimens pool, inclusion of recombinant protein sources can readily incorporate N antigens into the benchmarking panel with nonsynonymous mutations that may alter the analytical performance of the diagnostic test (18, 19, 27). The observation of higher sensitivity toward clinical samples may be a function of differing immunoassay availability of the antigen, whether due to minor conformational differences between the antigen source types or due to differences in reagent formulations between the MSD quantification assay and the rapid tests, favoring release and solubilization of antigen from more complex clinical samples in the rapid test buffers over the MSD assay buffer. Further experiments could help to determine what factors contribute to this observation.

### Conclusion.

The benchmarking panel allowed rapid assessment of the analytical performance by manufacturers and third parties in a manner that could be directly compared across RDTs for SARS-CoV-2. Full characterization of both molecular and protein analytes allowed for comparison of results across testing platforms. The benchmarking results were complementary to efforts to produce international standards and to support clinical evaluations.

## MATERIALS AND METHODS

### Materials.

Full-length recombinant N antigens expressed in E. coli and in HEK293 mammalian cells were purchased from Native Antigen (Kidlington, UK) and Acro Biosystems (Newark, DE, USA), respectively. Recombinant S antigen, in a stabilized trimeric form and expressed in HEK293 mammalian cells, was purchased from Acro Biosystems.

The following reagents were obtained through BEI Resources (Manassas, VA, USA) and the U.S. National Institute of Allergy and Infectious Diseases (NIAID)/U.S. National Institutes of Health (NIH), contributed by the U.S. Centers for Disease Control and Prevention (CDC): SARS-related coronavirus 2, isolate USA-WA1/2020, gamma-irradiated (NR-52287), and genomic RNA from SARS-related coronavirus 2, isolate USA-WA1/2020 (NR-52285). The following reagents were obtained through BEI Resources and NIAID/NIH: human coronavirus 229E (NR-52726) and human coronavirus OC43 (NR-52725). NR-52287 used for N antigen determination had assigned values for the concentration of infectious virus of 2.8 × 10^5^ 50% tissue culture infective dose (TCID_50_)/mL and RNA (4.1 × 10^9^ copies/mL), determined prior to inactivation. The viral RNA control for RT-PCR was prepared from USA-WA1/2020 (47.5 ng/mL total RNA with an estimated 1.84 × 10^7^ GEs/mL).

Using the GenBank sequence of the 419 amino acids as a full-length SARS-CoV-2 N protein (GenBank accession number QHO62115.1) with an additional TEV cleavage site and a polyhistidine tag, an extinction coefficient was calculated based on ProtParam (28) of Abs 0.1% (1 g/liter) of 0.959. This extinction coefficient was used to confirm stock protein concentration of recombinant SARS-CoV-2 antigen that was aliquoted and stored at −80°C.

The buffer diluent contained 1× phosphate-buffered saline (10 mM PBS, 2.7 mM potassium chloride, 137 mM sodium chloride, pH 7.4) with 1% bovine serum albumin (BSA), 1× PBS + 1% BSA. Negative swab pool diluent contains pooled discarded SARS-CoV-2 PCR-negative human nasal swabs eluted into 1× PBS.

### Clinical samples.

Deidentified nasal swab eluates were acquired from the Washington COVID-19 Biorepository (Seattle, WA, USA) or Boca Biolistics (Pompano Beach, FL, USA). Nasal swab eluates used in this study were prepared in either 1× PBS or clinical transport medium (Noble Biosciences, Gyeonggi-do, Republic of Korea). Nasal eluates were classified as positive or negative by PCR upon receipt in the COVID-19 Biorepository. Five SARS-CoV-2-positive nasal eluates were used in preparation of the panel, and 200 SARS-CoV-2-positive nasal eluates and 205 SARS-CoV-2 negative nasal eluates were used in the evaluation of the clinical performance of the N and S antigen detection assays.

### Quantification of SARS-CoV-2 N and S antigens using SARS-CoV-2 antigen immunoassays.

An immunoassay detecting SARS-CoV-2 N and S antigens was developed using the Meso Scale Discovery (MSD) platform (Meso Scale Diagnostics, Rockville, MD, USA), which uses electrochemiluminescence for detection. A monoclonal antibody pair for N antigen detection, MM08 and MM05, was sourced from Sino Biological (Beijing, People’s Republic of China). A monoclonal antibody pair for S antigen detection, L2355 and L2215, from sourced from Leinco Technologies (Fenton, MO, USA). The capture antibodies were labeled with biotin using the EZ-Link Sulfo-NHS-LC-LC biotinylation kit (ThermoFisher Scientific, Waltham, MA, USA), and the detector antibodies were labeled with SULFO-TAG(GOLD SULFO-TAG NHS-Ester, Meso Scale Diagnostics). Any unbound biotin or SULFO-TAG was removed using desalting columns (Zeba 40k MWCO, ThermoFisher Scientific). The concentrations of antibodies were measured at 280 nm via a spectrophotometer (NanoDrop 2000C, ThermoFisher Scientific), and concentration of detector antibody following labeling and desalting was assumed to be 90% recovery of the starting concentration. Standards were prepared from recombinant HEK293-expressed full-length SARS-CoV-2 N protein and stabilized trimeric S protein (Acro Biosystems).

### Clinical sample testing with SARS-CoV-2 antigen assays.

N and S assays were run separately, using 25 μL/well of the 0.5 μg/mL biotinylated capture antibody was used to coat a blocked SECTOR small spot streptavidin plate (Meso Scale Diagnostics). Analysis of signal and quantification of unknowns relative to the standard curve were conducted using Meso Scale Diagnostics’ Discovery Workbench 4.0 software. For quantification, standards and blank were fit with a four-parameter log logit fit with 1/y2 weighting. The lower limit of detection (LOD) was defined by the software’s curve fitting. The lower limit of quantification (LLOQ) was defined by the lowest concentration of standard with signal above the following: the limit of blank plus 10 times the standard deviation of the limit of blank (29). The upper limit of quantification (ULOQ) was defined by both software and a back-calculated recovery average of 100% ± 20%. Standard curves spanned 0.128 pg/mL to 50 ng/mL of N antigen (9), and 0.128 pg/mL to 1,250 pg/mL of S antigen.

The concentration of SARS-CoV-2 N antigen was measured in 405 residual nasal swab eluates, characterized by PCR at CLIA-registered clinical laboratories, collected in July through December 2020 in Washington state. The samples were selected across a range of Ct values from original testing, with selection biased toward higher Ct values to better test sensitivity of the antigen assay. Clinical samples either found or anticipated to be above the antigen quantification range were diluted either 5- or 20-fold to bring them into range, if volume allowed. Replicate well values for positives with a coefficient of variation greater than 20% were repeated.

### Molecular testing for SARS-CoV-2.

Viral RNA was extracted from samples using the QIAamp viral RNA mini kit (Qiagen, Valencia, CA, USA) according to the manufacturer’s instructions and eluted in 100 μL buffer. A quantitative RT-PCR (qRT-PCR) assay to estimate the SARS-CoV-2 GE/mL was developed using the N1 primer set developed by the CDC with primers and probe procured from Integrated DNA Technologies (Coralville, IA, USA). Each 20 μL final reaction volume contained 5 μL of 4× TaqPath one-step RT-qPCR Master Mix (ThermoFisher Scientific), 0.5 μL of probe (5 μmol/L), 0.5 μL each of forward and reverse primers (20 μmol/L), 8.5 μL of nuclease-free water, and 5 μL of nucleic acid extract. Amplification was performed on an Applied Biosystems 7300 real-time PCR instrument (ThermoFisher Scientific). Thermocycling conditions consisted of 15 min at 50°C, 2 min at 95°C, and 45 cycles of 3 s at 95°C and 30 s at 55°C. The cutoff for positive samples was less than 40 cycles. The median Ct values were used to determine the viral GE concentration using a standard curve from SARS-CoV-2 genomic RNA (Isolate USA-WA1/2020).

### Benchmarking panel.

Commercially sourced, full-length, His-tagged recombinant N protein as described above was used. Radiation-inactivated, cultured SARS-CoV-2 virus (BEI Resources, NR-52287) stocks were thawed and diluted into either buffer or negative swab pool. Serial dilutions were further made into diluent, and aliquots were frozen. Clinical nasal swab discards from five different individuals, positive for SARS-CoV-2 by qRT-PCR, were selected and combined. The combined samples were then serially diluted into negative swab pool, aliquoted, and frozen. The aliquots were tested by qRT-PCR to quantify viral GE/mL. A benchmarking panel composed of dilutions of recombinant proteins, inactivated viral lysate, and clinical specimen pool was then defined and applied to all diagnostic tests in this study. The panel members were characterized for N antigen concentration using the N antigen immunoassay, as well as qRT-PCR for the clinical specimen dilutions. The benchmarking panel is described in Table 2.

**TABLE 2:** Components of the benchmarking panel used in evaluation of rapid diagnostic tests to detect nucleocapsid and spike SARS-CoV-2 antigens^*a*^

Category	Antigen source	Diluent/matrix	No. of dilutions	N antigen concn range
Recombinant N	HEK293-expressed, His-tagged	1 × PBS, 1% BSA	10	0.1 to 50 ng/mL, 5,000 ng/mL
Recombinant N	E. coli-expressed, His-tagged	1 × PBS, 1% BSA	10	0.1 to 50 ng/mL, 5,000 ng/mL
Inactivated virus	Gamma-inactivated SARS-CoV-2 (202-WA-1)	1 × PBS, 1% BSA	8	0.05 to 10 ng/mL, representing 10^4^ to 10^7^ GE/mL
Inactivated virus	Gamma-inactivated SARS-CoV-2 (202-WA-1)	Negative swab pool	8	0.05 to 10 ng/mL, representing orders of 10^4^ to 10^7^ GE/mL
Clinical dilutions	PATH biorepository	Negative swab pool	16	0.025 to 225 ng/mL, representing orders of 10^3^ to 10^7^ GE/mL
Specificity	OC43 and 229E cultured viral lysates	1 × PBS, 1% BSA	1	Not detected; quantity of huCoV-specific N antigen not determined; 5,000 TCID_50_/mL OC43 and 10,000 TCID_50_/mL huCoV229E
Specificity	Diluent controls	Negative swab pool	1	Not detected
Specificity	Diluent controls	1 × PBS, 1% BSA	1	Not detected

a BSA, bovine serum albumin; GE, genome equivalent; PBS, phosphate-buffered saline; SARS-CoV-2, severe acute respiratory syndrome coronavirus-2; TCID_50_, 50% tissue culture infective dose.

### Rapid diagnostic tests.

Four EUA and/or EUL SARS-CoV-2 RDTs were evaluated using N antigen benchmarking panels: the Abbott BinaxNOW COVID-19 Ag card test (EUA), Abbott Panbio COVID-19 Ag rapid test device (EUL), LumiraDx SARS-CoV-2 Ag test (EUA), and SD Biosensor Standard Q COVID-19 Ag home test (EUL). The tests were assigned, in no particular order, identification numbers of RDT 1 through RDT 4, for the purpose of this publication, to deidentify specific results.

### Evaluation of rapid diagnostic tests with benchmarking panels.

Each RDT was run using five replicates for clinically derived and very high concentration recombinant panel members due to material volume limitations. Ten replicates were run for all other panel members. Panel member aliquots were thawed on ice and mixed gently. A pipetted volume of the panel mixed into the rapid test-specific extraction buffer simulated the extracted swab material. Thereafter, the instructions for the rapid tests were followed, and the diluted panel, at its final concentration in the extraction buffer, was added to the test according to instructions. All panel concentrations were run until two levels of decreasing concentrations were negative for all replicates. Visually read test results were scored for test line intensity by comparison to either a score card provided by the manufacturer or to a 20-point magenta-colored intensity score card, prepared by PATH. Lumira reader signal output was recorded electronically.

### Statistical analysis of detection limits using benchmarking data.

Two statistical models were developed to determine the relationships between (i) analyte concentration and RDT test line intensity and (i) analyte concentration and probability of RDT positivity. For (i), a sigmoid function was fit to the data of the form:

$$\text{Test line intensity}=\frac{\text{α}}{1+\text{exp}\left[(m-\log_{10}\text{conc})/s\right]\;}$$

where α, *m*, and *s* are parameters representing the shape of the sigmoid curve, and conc is the analyte concentration. This model was separately fitted to each data type (clinical dilution, inactivated virus culture, and recombinant), and the step was repeated for all four RDTs. For (ii), a simple logistic regression model was fitted to the data, where analyte concentration was the independent variable, and the binary RDT result (1 = positive, 0 = negative) is the dependent variable. A categorical factor representing the data type (clinical dilution, inactivated virus culture, recombinant protein) was included as a covariate to allow for data type-specific fitted curves. This step was repeated for all four RDTs. The models described in (i) and (ii) were fitted in a Bayesian framework using the R brms package (30). Noninformative Gaussian priors were used for the parameters, and the models were run for 5,000 iterations after a burn-in of 2,500 iterations. Convergence of chains was assessed using the R-hat statistic and visual checks. The final fitted lines and surrounding shaded areas represent the median and 95% credible of the expected values of the posterior predictive distributions.

### Simulation of detection of clinical samples by rapid diagnostic tests.

**FIG 8:**
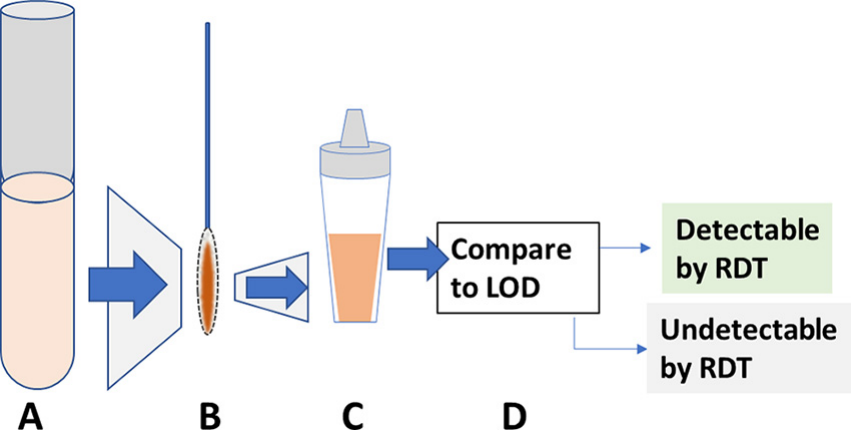
Representation of simulated clinical sample detection by rapid diagnostic test (RDT). (A) Clinical samples consisting of swab eluate in 3 mL transport medium; N antigen concentration is measured as pg/mL. (B) Total quantity of antigen assumed to be on swab equals concentration measured in A × 3 mL. (C) Total quantity of antigen is diluted in extraction buffer of RDT to calculate final concentration of N antigen added to RDT: quantity in B divided by total volume of RDT buffer. (D) Final concentration compared to 90% probability lower limit of detection from benchmarking to determine whether detectable by RDT. LOD, limit of detection.

The detection limits derived from benchmarking data were used to simulate the detection of clinical samples that had been characterized for SARS-CoV-2 N antigen concentration by MSD assay. For each clinical sample, the quantity of antigen, based on the measured N antigen concentration in 3 mL transport medium, was calculated to be instead diluted into the rapid test buffer, which is a smaller volume, thereby resulting in a higher concentration. Measured concentrations of N antigen per mL in 3 mL eluates was therefore multiplied by 3 to yield the total picograms of N antigen. This quantity of antigen was then divided by the manufacturer-designated volume of rapid test extraction buffer to simulate the final concentration of N antigen that would be added to the test. Finally, this final concentration was compared to detection limits generated in analysis of the benchmarking data to determine whether the sample would be designated detectable (Fig. 8).
